# The safety and performance of the Spectra Optia apheresis system platelet depletion protocol in patients with elevated platelet counts

**DOI:** 10.1002/jca.22009

**Published:** 2022-09-14

**Authors:** Pamela Lopert, Sohair Abdelrahman, Christopher A. Graybill, Jack Rhodes, Betina Sørensen, Ming Li, Yu Hu, Depei Wu, Ligen Liu, Pengcheng He, Xuejun Zhang, Fen Huang, Jianda Hu, Jerry Bill

**Affiliations:** ^1^ Terumo BCT, Inc. Lakewood Colorado USA; ^2^ Aarhus University Hospital Skejby Aarhus Denmark; ^3^ The Second People's Hospital of Shenzhen Shenzhen China; ^4^ Wuhan Union Hospital Wuhan China; ^5^ The First Affiliated Hospital of Soochow University Suzhou China; ^6^ Tongren Hospital Shanghai Jiaotong University School of Medicine Shanghai China; ^7^ The First Affiliated Hospital of Xi'an Jiaotong University Xi'an China; ^8^ The Second Hospital of Hebei Medical University Xi'an China; ^9^ Nanfang Hospital Guangzhou China; ^10^ Fujian Medical University Union Hospital Fujian China

**Keywords:** collection efficiency, essential thrombocythemia, myeloproliferative neoplasm, platelets, therapeutic apheresis, thrombocytapheresis, thrombocytosis

## Abstract

**Background:**

Thrombocytosis is a presenting and progressive clinical feature found in multiple disease states. It is characterized by high platelet (PLT) counts (>450 × 10^9^/L) and can lead to thrombohemorrhagic events. Thrombocytapheresis or platelet depletion (PLTD) can be performed in acutely symptomatic patients suffering from thrombocytosis and may reduce or prevent acute serious complications associated with thrombocythemia thereby enabling patients to receive potentially curative high‐dose chemotherapy.

**Methods:**

This report details the results from 2 clinical studies, one conducted in the European Union (EU) and one in the People's Republic of China, assessing the PLTD procedure on the Spectra Optia Apheresis System. The primary objective of both studies was to assess the safety and performance of the PLTD procedure in patients with elevated PLT counts.

**Results:**

Data were collected from 56 participants completing 64 PLTD procedures. The mean percent change in PLT count and collection efficiency (CE1) was 55.1% and 68.5%, respectively. In the EU study, 6 participants experienced a total of 9 adverse events (AEs) and in the China study, 44 participants reported a total of 212 AEs. In both studies, the majority of AEs reported were Grade 2 or lower and no serious AEs, unanticipated adverse device effects, or AEs leading to death were reported.

**Conclusions:**

The data collected within these studies indicate that the PLTD procedure is well tolerated and effective at reducing circulating PLTs in patients suffering from thrombocytosis as evaluated by a percent decrease in PLT count, CE1, and AE incidence.

## 1 INTRODUCTION

Myeloproliferative neoplasms (MPNs) are a group of blood disorders that occur as a result of improper development of blood cells within the bone marrow, including red blood cells (RBC), platelets (PLT), and white blood cells (WBC). Patients suffering from MPNs may present with leukocytosis (high WBC count) and/or thrombocytosis (high PLT count), with symptoms ranging from minor to life‐threatening.^1^ Essential thrombocythemia (ET) is a rare, chronic MPN diagnosed in approximately 2 to 3 per 100,000 individuals annually.^2^, ^3^ In ET, PLT counts may exceed 1000 × 10^9^/L (normal range 150‐450 × 10^9^/L).^4^ These excessively high PLT counts may lead to thrombosis, which can obstruct blood vessels and result in stroke, myocardial infarction, or pulmonary embolism. In some patients, high PLT counts may also cause hemorrhage as a result of subsequent inadequate levels of von Willebrand factor.

Thrombocytapheresis, or platelet depletion (PLTD) by apheresis, is performed in acutely symptomatic patients suffering from thrombocytosis often attributable to MPNs such as ET, polycythemia vera, chronic myeloid leukemia (CML), and myelofibrosis. While thrombocytapheresis procedures are not curative, they may reduce or help prevent potentially serious complications associated with high PLT counts thereby enabling patients to receive potentially curative high‐dose chemotherapy. Thrombocytapheresis should be considered in patients who are poor responders or have a contraindication to drug therapy and in emergent events as a bridging treatment where rapid PLT reduction is needed.

Data on the use of apheresis in the treatment of individual diseases and disorders is often limited because randomized, controlled apheresis trials have not been performed for most conditions. As such, the American Society for Apheresis (ASFA) has created guidelines on the use of therapeutic apheresis in order to summarize the most current literature on the use of apheresis in treating diseases, to provide a critical review of this literature, and to give practical guidance to apheresis practitioners.^5^ The ASFA guidelines indicate a potential benefit of thrombocytapheresis in the treatment of patients with thrombocytosis, as case studies have described rapid improvement of severe microvascular ischemic or hemorrhagic complications that are not responsive to antiplatelet therapies.^5^

This report details the results from two clinical studies, one conducted in the European Union (EU) and one in the People's Republic of China, that assessed the use of the Spectra Optia Apheresis System (hereon referred to as Spectra Optia) for thrombocytapheresis in patients with thrombocytosis. The main objective of both studies was to show that the PLTD protocol conducted on the Spectra Optia could safely remove PLTs in patients with elevated PLT counts.

## 2 MATERIALS AND METHODS

### 2.1 Study conduct

This report outlines the results from two multicenter, single‐arm studies which assessed the use of the PLTD protocol on Spectra Optia (Terumo BCT, Lakewood, CO) in patients with elevated PLT counts. The first study (NCT02308787) was a retrospective data collection study to evaluate the routine use, performance and safety of the PLTD procedure at two European sites (Aarhus University Hospital in Denmark and the University of Pécs in Hungary). The second study was a prospective trial to characterize the performance and safety of a single PLTD procedure in patients with a PLT count ≥1000 × 10^9^/L at eight sites in the People's Republic of China (The Second People's Hospital of Shenzhen, Wuhan Union Hospital, Tongren Hospital Shanghai Jiaotong University School of Medical, The First Affiliated Hospital of Xi'An Jiaotong University, The Second Hospital of Hebei Medical University, The First Affiliated Hospital of Soochow University, Nanfang Hospital, and Fujian Medical University Union Hospital).

Device performance and safety data were collected both prospectively and retrospectively. Data collected pre‐procedure included medical history, demographics, diagnoses, symptoms, vital signs, laboratory measurements (eg, complete blood count [CBC]), concomitant medications, and the type and volume of blood products or medications administered the day of the procedure. During and following each procedure, data were collected for basic procedural and device information, clinical laboratory measurements from both the participant and the depletion product (waste bag) if available, blood products, medications, or replacement fluids required, and adverse events (AE), and device deficiencies.

The study protocol was approved by ethics committees (EC) at all sites and adhered to the principles that have their origins in the Declaration of Helsinki, the International Conference on Harmonization Guidelines for Good Clinical Practice, and the International Organization for Standardization (ISO) International Standard 14 155:2011. For the retrospective study, written informed consent was waived by local ECs as no participant identifying data was collected. For the prospective study, all participants signed the EC‐approved informed consent form prior to any study procedure being conducted.

### 2.2 Selection and description of participants

For the retrospective study, medical records between November 2011 through May 2014 were screened for patients who had received a minimum of one PLTD procedure on the Spectra Optia and had available pre‐ and post‐procedure PLT counts. For the prospective study (conducted from August 2019 through November 2020), adult participants with a PLT count ≥1000 × 10^9^/L and with adequate venous access were eligible to enroll. Participants must not have had (1) any significant bleeding in the 24 hours prior to enrollment as determined by the physician, (2) undergone a thrombocytapheresis procedure in the previous 7 days, (3) a hypersensitivity to anticoagulants, and (4) for female participants, they could not be pregnant or lactating. Patient demographics and study endpoint data are presented for each study individually and combined in tabular format. Participants in both studies underwent a thrombocytapheresis procedure on the Spectra Optia due to thrombocytosis and are therefore representative of the patient population of interest.

### 2.3 Clinical endpoints

Study endpoints included the percent decrease in PLT counts in participant blood following PLTD procedure(s), the percent of processed PLTs collected (ie, collection efficiency [CE1] for PLTs), and AEs observed within 2 hours (retrospective study) and 24 hours (prospective study) post‐procedure. Adverse events were defined per ISO 14 155 Section 3.2 as any untoward medical occurrence, unintended disease or injury, or untoward clinical signs (including abnormal laboratory findings) in participants, users or other persons, whether or not related to the investigational medical device and whether anticipated or unanticipated. Additionally, investigators were asked to judge whether or not each AE was related to the thrombocytapheresis procedure, the Spectra Optia device, and/or participant's medical history (prospective study only). Device‐related AEs were defined as any AE which, in the opinion of the investigator, would only occur due to the use of the Spectra Optia and would not occur on other therapeutic apheresis devices.

All AEs were graded using the National Cancer Institute Common Terminology Criteria for Adverse Events (CTCAE), Version 4.03, and coded using the Medical Dictionary for Regulatory Activities (MedDRA). Device deficiencies were defined per ISO 14155:2011 Section 3.15 as an inadequacy of a medical device with respect to its identity, quality, durability, reliability, safety or performance, and includes malfunctions, use errors and inadequate labeling.

The calculation for CE1 (see below) incorporated pre‐ and post‐apheresis PLT counts thereby accounting for PLT removal via the procedure and PLT mobilization during the procedure. For both studies, the apheresis parameters were determined according to the Spectra Optia Apheresis System Operator's Manual and site SOPs. For the prospective study, the run target was set at a minimum of 1.5 total blood volume (TBV) and the only anticoagulant allowed was ACD‐A. Replacement fluid, if required, was administered per institutional standard of care in both studies.

The Spectra Optia Apheresis System was used for all PLTD procedures and is comprised of (1) hardware which consists of the actual apheresis machine and an associated removable centrifuge filler, (2) a sterile single‐use disposable tubing set (the Spectra Optia IDL set), and (3) embedded software for PLTD procedure.

### 2.4 Statistics

At least 10 participants were prespecified as needed for the retrospective study and up to 44 participants (to get 34 evaluable procedures) for the prospective study. Analyses were conducted using SAS (SAS Institute, Cary, North Carolina) software Version 9.3 and 9.4. Primary analysis of performance was conducted using the Full Analysis Set (FAS), which consisted of participants who completed the depletion procedure with all primary endpoint measurements. Performance endpoints were calculated as described below.

The primary endpoint, the percent decrease in PLT count, was calculated as:

$$∆=100\times \frac{{\mathrm{PLT}}_{\mathrm{pre}}-{\mathrm{PLT}}_{\text{post}}}{{\mathrm{PLT}}_{\mathrm{pre}}}$$

The secondary endpoint, CE1, was calculated as:

$$\mathrm{CE}1=\frac{{\mathrm{PLT}}_{\text{collected}}\times \text{Volume}\;{\mathrm{mL}}_{\text{collected product}}}{\frac{{\mathrm{PLT}}_{\mathrm{pre}}+{\mathrm{PLT}}_{\text{post}}}{2}\times \text{Volume}\;\mathrm{mL}\;\left(\text{blood processed}\right)}$$

Safety data were collected using the Safety Set (SS) which comprised of all participants who initiated a PLTD procedure. Data were summarized with the mean, SD, median, and range for continuous variables and with frequencies and percentages for discrete variables.

## 3 RESULTS

### 3.1 Participant disposition and demographics

Data were retrospectively collected from 12 participants who underwent 20 PLTD procedures and prospectively from 44 participants who underwent 44 PLTD procedures for a total of 56 participants completing 64 PLTD procedures. In the retrospective study, the majority of patients (8 [66.7%]) underwent 1 PLTD procedure while 3 participants (25.0%) had 2 procedures and 1 participant (8.3%) underwent 6 PLTD procedures. In the prospective study, all 44 participants underwent 1 PLTD procedure.

Individual study and cumulative participant demographics data are presented in Table 1. The majority of participants were female (58.9%) with a mean age and BMI of 56.2 ± 17.44 years (21‐90 years) and 22.8 ± 2.97 kg/m^2^ (17‐30), respectively. The majority of patients were diagnosed with ET (66.1%) followed by thrombocytosis (12.5%), CML (8.9%) and myelodysplastic/myeloproliferative neoplasm (5.3%). Participant demographics were similar between studies.

**TABLE 1:** Participant demographics and baseline disease characteristics

Characteristic	Retrospective (n = 12)	Prospective (n = 44)	Total (N = 56)
Age (years)			
Mean (SD)	64.3 (13.0)	54.0 (17.96)	56.2 (17.44)
Range	42–77	21–90	21–90
Sex, n (%)			
Female	8 (66.7)	25 (56.8)	33 (58.9)
Male	4 (33.3)	19 (43.2)	23 (41.1)
Height (cm)			
Mean (SD)	163.9 (9.22)	163.5 (8.09)	163.6 (8.25)
Range	148‐181	149‐180	148–181
Weight (kg)			
Mean (SD)	63.6 (13.41)	60.84 (1.53)	61.4 (11.88)
Range	47‐88	41‐85	41‐88
BMI			
Mean (SD)	23.4 (3.03)	22.6 (2.96)	22.8 (2.97)
Range	18‐30	17‐29	17–30
Total blood volume (L)^a^			
Mean (SD)	4.0 (0.7)	3.9 (0.7)	4.0 (0.7)
Range	3.1‐5.6	2.7‐5.4	2.7‐5.6
Diagnosis, n (%)			
Essential thrombocythaemia	9 (75.0)	28 (63.6)	37 (66.1)
Thrombocytosis	0	7 (15.9)	7 (12.5)
Chronic myeloid leukaemia	1 (8.3)	4 (9.1)	5 (8.9)
Myelodysplastic/myeloproliferative neoplasm	1 (8.3)	2 (4.5)	3 (5.3)
Polycythaemia vera	1 (8.3)	0	1 (1.8)
Chloroma	0	1 (2.3)	1 (1.8)
Myelofibrosis	0	1 (2.3)	1 (1.8)
Primary myelofibrosis	0	1 (2.3)	1 (1.8)

Abbreviations: cm, centimeters; kg, kilograms; L, liter(s); N or n, number; SD, standard deviation.

^a^ Estimated by Nadler's formula for total blood volume based on gender, height, and weight.

### 3.2 Procedure characteristics and device performance

A summary of the procedure characteristics and device performance results is presented in Table 2. The mean duration of the PLTD procedures was 162.4 ± 25.0 minutes, with a maximum procedure time of 221 minutes. Pre‐procedure PLT counts were expectedly high for all participants with an average PLT count of 1534.7 ± 580.3 × 10^9^/L which ranged from 867 × 10^9^/L to 4185 × 10^9^/L. All pre‐procedure PLT counts were well above the normal range of 150 to 450 × 10^9^/L.

**TABLE 2:** Platelet depletion characteristics and device performance

No. participants	Retrospective (*N* = 12)	Prospective (*N* = 43)	Total (*N* = 55)
No. PLTD Procedures	20	43	63
No. Total blood volumes processed			
Mean (SD)	1.7 (0.2)	1.5 (0.0)	1.6 (0.16)
Range	1.4‐2.2	1.5	1.44‐2.25
Whole blood processed (mL)			
Mean (SD)	6652.3 (856.4)	5903.3 (1074.16)	6141.1 (1062.98)
Range	5130‐9000	4112‐8124	4112‐9000
Run Time (min)			
Mean (SD)	144.5 (17.81)	170.8 (23.55)	162.4 (25.01)
Range	113‐185	128‐221	113‐221
Total waste bag volume^a^ (mL)			
Mean (SD)	669.8 (248.5)	529.3 (177.63)	557.9 (221.66)
Range	333‐1445	289‐1271	289‐1445
PLT count (pre‐procedure; 10^9^/L)			
Mean (SD)	1740.7 (745.0)	1438.9 (465.30)	1534.7 (580.33)
Range	867‐4185	1001‐3022	867‐4185
PLT count (post‐procedure; 10^9^/L)			
Mean (SD)	717.2 (336.0)	643.88 (209.21)	667.1 (255.79)
Range	309‐1630	299‐1037	299‐1630
PLT count (% change)			
All Procedures (n)	20	43	63
Mean (SD)	58.2 (12.0)	53.6 (4.95)	55.1 (14.16)
Range	16‐73	10‐80	10‐80
CE1 for PLTD (% processed)^a^			
All Procedures (n)	11	43	54
Mean (SD)	67.8 (18.3)	68.66 (18.89)	68.48 (18.60)
Range	28‐92	26‐120	26‐120
Hematocrit (% change)			
All Procedures (n)	12	43	55
Mean (SD)	6.2 (17.0)	8.51 (5.87)	8.0 (9.31)
Range	−25.8 to 25.0	−2.7 to 30.4	−25.8 to 30.4
WBC count (% change)			
All Procedures (n)	17	43	60
Mean (SD)	25.4 (16.99)	17.9 (15.62)	20.0 (16.24)
Range	−4.9 to 60.1	−33.3 to 45.4	−33.3 to 60.1

Abbreviations: CE1, collection efficiency; min, minute; mL, milliliter; No, number; PLT, platelet; PLTD, platelet depletion; WBC, white blood cell.

^a^ One site did not collect data from the waste bag; therefore retrospective data presented is only from 11 procedures.

The mean percent change in participant PLT counts post‐procedure was 55.1 ± 14.2%, with a maximum decrease of 80%. The mean CE1 of the PLTD procedures as measured from the depletion product (waste bag) contents was 68.5 ± 18.6% with a maximum CE1 of 120%. To note, the CE1 results are from a total of 54 procedures as 1 site in the retrospective study did not collect PLT counts from the waste bag.

### 3.3 Safety

The AE reporting timeframe was different between studies (from start of the apheresis procedure until discharge from the apheresis unit in the retrospective study and from the start of the procedure until 24‐hour post‐procedure in the prospective study), therefore, AE data were not combined and is reported per study in Table 3. In the retrospective study, 6 participants (50.0%) experienced a total of 9 AEs and in the prospective study, 44 participants (100%) reported a total of 212 AEs. In both studies, there were no AEs related to the Spectra Optia device, no SAEs, no unanticipated adverse device effects, and no AEs leading to death reported.

**TABLE 3:** Adverse events

	Retrospective (n = 12)	Prospective (n = 44)
Total number of AEs, n	9	212
Participants with at least 1 AE, n (%)	6 (50.0)	44 (100.0)
Device related AEs	0	0
Procedure related AE	5 (41.7)	18 (40.9)
Medical history related AEs	NR	19 (43.2)
SAEs	0	0
UADE	0	0
AE leading to discontinuation	1 (8.3)	0
AE leading to death	0	0
Preferred Term occurring in >10% of population, n (%)		
Anemia	3 (25.0)	12 (27.3)
Protein total decreased	0	12 (27.3)
Prothrombin time prolonged	0	9 (20.5)
Blood fibrinogen decreased	0	8 (18.2)
Neutrophil percentage increased	0	8 (18.2)
Hypophosphataemia	0	8 (18.2)
WBC decrease	1 (8.3)	6 (13.6)
Hypomagnesaemia	0	7 (15.9)
Blood albumin decreased	0	6 (13.6)
Globulins decreased	0	6 (13.6)
Lymphocyte count decreased	0	6 (13.6)
Monocyte percentage decreased	0	6 (13.6)
Platelet‐large cell ratio decreased	0	6 (13.6)
Thrombin time prolonged	0	5 (11.4)
Hypoalbuminaemia	0	5 (11.4)
Hypotension	3 (25.0)	1 (2.3)

Abbreviation: NR, not reported.

In the retrospective study, anemia and hypotension (25.0% each) were the only AEs reported in more than 1 participant. Of the 9 reported AEs, 5 were Grade 1, 3 were Grade 2, and 1 was Grade 3. The 1 Grade 3 AE was a WBC count decrease based on the post‐procedure WBC count (1.3 × 10^9^/L) which was a 41.4% decrease from the pre‐procedure count. This participant was receiving concurrent myelosuppressive therapy for their underlying disease (CML) which may account for the WBC decrease. Seven of the nine AE were reported to be related to the procedure with the exception of two Grade 2 anemias reported in two participants. One participant terminated their procedure prematurely due to a Grade 1 AE of hypotension. This AE was considered to be related to the study procedure but no further action (eg, administration of concomitant medications or replacement fluids) was required to treat the hypotension and the AE was resolved.

In the prospective study, the most common AEs included anemia (12 AEs in 12 participants), total protein total decreased (12 AEs in 12 participants), and prothrombin time prolonged (9 AEs in 9 participants). Eighteen participants had 88 AEs related to only the PLTD procedure, 19 participants had 40 AEs related to their medical history and 9 participants reported 28 AE related to the study device. The majority of AEs (184 AEs from 26 subjects) were reported as mild/Grade 1. Twenty‐one AEs (from 12 subjects) were considered moderate/Grade 2, and 7 AEs from 6 subjects were severe/Grade 3.

WBC and RBC loss, which are anticipated laboratory changes following a PLTD procedure, were observed in both studies. Most of the AEs reported were related to hematologic and blood chemistry abnormalities (eg, anemia, total protein decrease, and WBC decrease). The mean post‐procedure WBC count and hematocrit percent were 12.2 ± 13.0 × 10^9^/L and 34.8 ± 8.8%, respectively, with a mean WBC and hematocrit percent loss of 20.0 ± 16.2% and 8.0 ± 9.3%, respectively. To note, most post‐procedure WBC counts decreased but remained within the normal range (3.5‐10.5 × 10^9^/L). There were 3 patients who had post‐procedure counts below the normal range, and all three had pre‐counts at or below the normal range. Regarding RBC loss, the average pre‐procedure hematocrit was 34.1 ± 8.8% (regardless of sex) which is below the normal range and the average percent decrease of hematocrit was 8.0 ± 9.3%. Anemia was reported for 15 participants and none were considered serious.

## 4 DISCUSSION

This study reports performance and safety outcomes from both a routine use and a prospective clinical trial assessing the PLTD procedure conducted on the Spectra Optia. Patients who had an elevated PLT count (>450 × 10^9^/L) and needed to have their PLT count rapidly reduced to prevent potentially serious complications, were enrolled in these studies. The criteria for a PLTD procedure were determined by the treating physician's judgment of the patient's PLT counts and their overall clinical status. Results from both the retrospective and prospective studies show the PLTD procedure can provide the anticipated clinical performance as indicated by a 55% PLT count decrease and a mean CE1 of 69%.

According to the ASFA guidelines, a PLTD apheresis procedure can be expected to lower a patient's PLT counts by 30% to 60% and should be conducted daily or as indicated to reach/maintain a PLT count goal as defined by the treating physician.^5^ Results from both the retrospective and prospective studies are consistent with these guidelines (reduction of 58.2 ± 12.0 and 53.6 ± 4.95, respectively), demonstrating that a PLTD procedure conducted on the Spectra Optia can provide the anticipated clinical benefit consistent with reported literature and practice guidelines.

As PLT counts in the waste bag are not routinely measured, CE1 data for PLTD procedures are not routinely reported in the literature and is generally not used clinically. However, this measurement may be of use clinically in cases where little to no decrease in PLTs is observed following a PLTD procedure, as it could help distinguish between device performance and mobilization/production of PLTs during the procedure. It has been documented during thrombocytapheresis procedures in acute leukemia patients that there is a replenishment of the circulating platelet pool by the spleen in response to the rapid removal of PLTs.^6^ To help address mobilization, the CE1 can be utilized to determine the total number of PLTs collected to determine the device performance vs participant PLT mobilization. Within the two studies reported in this paper, PLT counts were obtained from the waste bags in the majority (84.4%) of procedures and the mean CE1 of the PLTD procedures was 68.5 ± 18.6%. The CE1 and decreases in PLT counts were consistent across studies reported here indicating the PLTD procedure on the Spectra Optia device is efficient in PLT removal in clinical practice in a wide variety of patients and geographical locations.

Most literature to date has only reported case report or case study data on patients with elevated PLT counts undergoing a PLTD procedure.^5^, ^7^, ^8^, ^9^ These reports have described an improvement in symptoms and thrombotic complications in patients who have been unresponsive or who cannot undergo cytoreductive or other first‐line therapies (ie, pregnant women). ASFA has recommended thrombocytapheresis as a second‐line therapy for thrombocytosis, either as a standalone treatment or in conjunction with other modes of treatments.^5^ More recently, a few clinical trials have been conducted assessing the efficacy of thrombocytapheresis adjunct to pharmacotherapy and have demonstrated improved clinical outcomes.^10^, ^11^ Baron, et al., retrospectively analyzed the combined use of thrombocytapheresis and chemotherapy for symptomatic thrombocytosis in 29 MPN patients. All patients achieved remission within an average range of 0.6 to 2.6 months following initial combination treatment.^11^ As expected, thrombocytapheresis was effective in lowering PLT counts and reversing symptoms quickly and provided protection against new or progressive complications while the patient was able to undergo the myelosuppressive therapy they needed for long‐term treatment of their MPN.^11^

Nguyen et al. retrospectively analyzed data from MPN patients who underwent a single thrombocytapheresis procedure on the Spectra Optia prior to initiation of chemotherapy.^10^ They found after a single procedure patients had a 44.5% median reduction in PLT count and a median CE1 of 65.2%.^10^ The values reported by Nguyen et al. are similar to the results obtained in this study, indicating that thrombocytapheresis can reduce circulating PLT counts in symptomatic MPN patients.

The frequency and type of AEs observed in these studies are reflective of the clinically fragile nature of this patient population, and no observed safety signals were attributed to the Spectra Optia device. The most common AEs reported were related to decreases in hematologic parameters and blood chemistry abnormalities. It is anticipated for patients to lose a non‐clinically significant number of WBCs and RBCs from a thrombocytapheresis procedure due to the presence of these blood components at the PLT collection interface. MPN patients often have anemia and elevated WBC counts (which was observed in the patient population in this report) and although decreases in RBCs and WBCs are a known side effect of thrombocytapheresis, the average percent decrease of hematocrit in this study was <9% and the majority maintained a WBC count in normal range.^12^ As anemia is a known side‐effect of MPNs treatment, it can be managed either prior to the apheresis procedure or after with pharmaceutical interventions or an RBC transfusion. There were no Grade 4/5 AE, SAEs, or AEs related to the device reported in either study indicating the PLTD procedure conducted on the Spectra Optia is safe and the majority of AEs are related to the underlying disease.

## 5 CONCLUSIONS

The data collected within these studies indicates that the PLTD procedure using the Spectra Optia is well tolerated and efficacious at decreasing circulating PLTs in patients experiencing thrombocytosis as evaluated by the percent decrease in PLT count, the CE1 for PLTs, and the AE incidence.

## FUNDING INFORMATION

Terumo BCT provided funding for these studies.

## CONFLICT OF INTEREST

Pamela Lopert, Sohair Abdelrahman, Christopher A. Graybill, Jack Rhodes, and Jerry Bill were, at the time of the studies, employees of Terumo BCT. The institutions that conducted these clinical trials received support from Terumo BCT.

## ETHICS STATEMENT

Clinical study protocols were approved by ethics committees (EC) at all sites and adhered to the principles that have their origins in the Declaration of Helsinki, the International Conference on Harmonization Guidelines for Good Clinical Practice, and the International Organization for Standardization International Standard 14155:2011(E).

## PATIENT CONSENT STATEMENT

Written informed consent was waived by local Ethics Committees for the European retrospective study as no participant identifying data was collected. For the prospective study, all participants signed the Ethics Committee‐approved informed consent form prior to any study procedure being conducted.

## PERMISSION TO REPRODUCE MATERIAL FROM OTHER SOURCES

There are no reproductions of material from other sources presented in this article.

## CLINICAL TRIAL REGISTRATION

The retrospective data collection study was registered on Clinicaltrials.gov under NCT02302365. The prospective study was not registered as it did not meet the criteria for registration per Applicable Clinical Trial (ACT) Under 42 CFR 11.22(b).

## Data Availability

Data supporting the findings of this study are available within the article.
